# Early therapeutic efficacy of condoliase chemonucleolysis for lumbar disc herniation

**DOI:** 10.1186/s13018-024-05405-4

**Published:** 2024-12-30

**Authors:** Kazuyoshi Kobayashi, Koji Sato, Yoshinori Morita

**Affiliations:** Department of Orthopaedic Surgery, Japan Red Cross Aichi Medical Center Nagoya Daini Hospital, Myokencho 2-9, Syowa-Ku, Nagoya, Aichi 466-8650 Japan

**Keywords:** Condoliase, Lumbar disc herniation, Chemonucleolysis, Leg pain, Low back pain, Early therapeutic efficacy

## Abstract

**Background:**

Low back pain is often caused by lumbar disc herniation (LDH). Treatment of LDH is possible using chemonucleolysis of the nucleus pulposus with condoliase injection. However, onset of the therapeutic effect varies among patients, with improvement from an early stage to 3 months post-injection. This study was performed to identify the characteristics of early responders to condoliase therapy.

**Methods:**

A retrospective cohort study was performed in 371 consecutive patients (259 males, 112 females; age, 49.9 ± 18.7 years; follow-up period, 13.1 ± 7.4 months) treated with condoliase injection for LDH between August 2018 and January 2024. Chemonucleolysis was performed with 1 mL of condoliase (1.25 U/mL) injected into the intervertebral nucleus pulposus. Clinical assessments were made before injection and 1 day, and 1, 4 and 12 weeks post-injection. Pain was measured on a visual analogue scale. Herniation parameters were evaluated on axial MRI. The herniated disc volume was measured on plain lumbar radiography. Demographic and clinical data were taken from medical charts. Multivariate logistic regression analysis was used to identify factors with independent relationships with treatment efficacy.

**Results:**

Improvement of leg pain from baseline by ≥ 50% occurred in 21% of cases within one day after condoliase treatment. Patients with this improvement at 1 week post-injection were defined as early responders (n = 142, 38.3%). In multivariate analysis, age < 40 years (*p* = 0.022, odds ratio (OR): 1.71, 95% confidence interval (CI): 1.12–4.35), Pfirrmann Grade II or III at baseline (*p* = 0.032, OR: 1.86, 95% CI: 1.17–5.41), and a high intensity MRI signal in the herniation (*p* = 0.041, OR: 1.87, 95% CI: 1.06–5.27) were significantly associated with early improvement. No patients had anaphylactic shock or neurologic sequelae.

**Conclusions:**

This study confirms the safety and efficacy of chemonucleolysis with condoliase for treating patients with painful LDH. Age, high-intensity MRI signals, and baseline Pfirrmann grade were significant factors associated with early improvement.

## Background

Low back pain is a frequent symptom encountered in clinical practice. This condition if often due to lumbar disc herniation (LDH), which is a common degenerative discogenic disease. Patients with LDH often report radiating leg pain, in addition to low back pain. While 60–80% of patients have spontaneous improvement in symptoms within 6–12 weeks [1], LDH can severely affect quality of life. Thus, effective management of this condition is important.

Chemonucleolysis is used to treat LDH using enzymatic elimination of the nucleus pulposus in an intervertebral disc [2]. This is a less invasive method that is positioned between conservative treatment and surgery [2]. Initially introduced by Smith [3], chemonucleolysis with chymopapain became a widely adopted treatment for LDH in the late twentieth century, with reports of good clinical outcomes [4, 5]. However, the rare but serious side effects of chymopapain, including fatal anaphylactic reactions, led to discontinuation of its use in 2002 [5]. Condoliase (chondroitin sulfate ABC endolyase) was launched in August 2018 in Japan as an enzyme for chemonucleolysis with improved safety and efficacy [6, 7]. Chondroitin sulfate and hyaluronic acid (glycosaminoglycans in the nucleus pulposus) are particularly good substrates of condoliase [8], and the reported effectiveness of condoliase is 62–85% with no major adverse events [9–14].

Despite the success of condoliase, the time of onset of therapeutic effects varies among individuals and the treatment is not characterized by immediate pain relief. This is because the mechanism of action is chemonucleolysis, rather than direct pain relief. Onset of therapeutic effects generally occurs after about 3 months, but some patients have earlier symptomatic improvement and favorable outcomes. However, no studies have focused on the timing of therapeutic effects, particularly in cases that respond early to treatment. Thus, the goal of this study was to examine the characteristics of cases with an early response to condoliase therapy.

## Materials and methods

### Study population

The study included 371 consecutive patients with LDH who received intradiscal injection of condoliase at our clinic between August 2018 and January 2024. Among these cases, 24 received surgery in the post-injection follow-up period. The inclusion criteria were cases indicated for intradiscal injection of condoliase, including lower-extremity pain on one side, compression of a nerve root due to disc herniation disc shown on MRI, neurological signs indicating distribution of the compressed nerve root, and lack of responsiveness to conservative treatment. The exclusion criteria were motor weakness (score < 4 on a manual muscle test); cauda equina syndrome, developmental spine deformity, or a history of discectomy at the same level as the intradiscal injection. For patients eligible for both surgery and injection, we adopted a shared decision-making process that considered the clinical profile and preferences of the patient, and the less invasive nature of the injection compared to surgery. The protocol was approved by the Human Ethics Review Committee of our Medical Faculty and followed the Clinical Research Guidelines of the Ministry of Health, Labor, and Welfare of the Japanese Government. All patients provided written informed consent. The study was performed as a retrospective cohort study.

### Procedure

Chemonucleolysis was conducted or overseen by an experienced orthopedic spine surgeon, with the patient in a semi-lateral decubitus position. The imaging arm was adjusted to provide parallel visualization of the adjacent endplates of the disc. Using a posterior-lateral approach, a 21-gauge disc-puncture needle was inserted from the contralateral side of the herniation into the intervertebral disc under local anesthesia and fluoroscopic guidance. Condoliase (1.25 U/mL) was injected (1 mL) toward the middle of the affected intervertebral nucleus pulposus [7, 15]. Patients were observed for 3 h post-injection and then returned home without use of prophylactic antibiotics.

### Clinical evaluation

Radiological assessments were made prior to the injection. Clinical status was assessed before the injection and at 1 day, and 1, 4 and 12 weeks post-injection. Pain intensity was evaluated on a visual analog scale (VAS), with 0 indicating no pain and 10 indicating the worst pain experienced. Cases were identified with leg pain improvement of ≥ 50% at each assessment timepoint post-injection compared to baseline [7, 15] Data for demographics and clinical factors were obtained from medical charts for age, gender, body mass index (BMI), intervertebral disc level, symptomatic period pre-injection, Pfirrmann criteria, classification of herniation, MRI high-intensity signal in the herniation on T2-weighted sagittal and axial images [9], area of injection of the intervertebral disc, bulging ratio of herniation, herniated mass volume, and adverse events. Cases with leg pain improvement of ≥ 50% at 1 week post-injection were placed in the early responders group (group E), with all others in the non-early responders group (group non-E) (Fig. 2). Group non-E includes the 24 patients who underwent surgery. Additional groups were defined by age (≥ 40 vs. < 40 years), with the 40 years cutoff based on previous MRI findings [16].

### Radiographic assessment

**Fig. 1:**
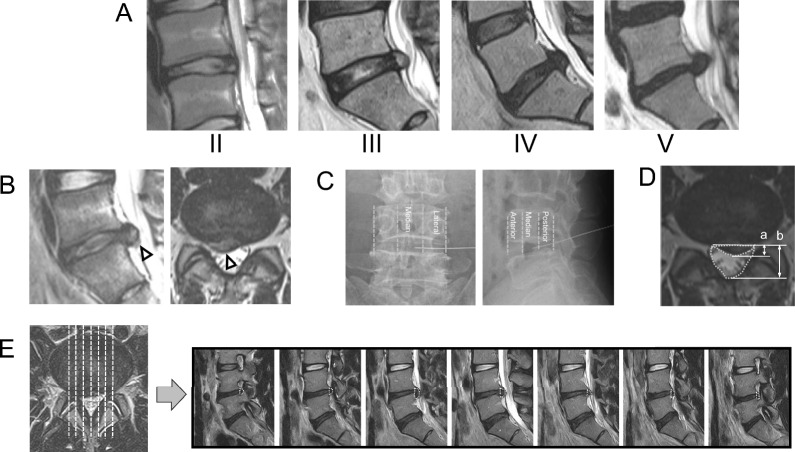
**A**: Degree of disc degeneration using Pfirrmann criteria. **B**: High intensity MRI signal in the herniation. **C**: Injection region. **D**: Bulging ratio of herniation (a/b × 100%; a: anteroposterior diameter of herniation, b: anteroposterior diameter of canal space). **E**: Measurement of herniated mass volume

Axial and sagittal MRI at baseline was used to classify cases into those with herniations of the sub- and trans-ligamentous extrusion types [17] and to determine the level of intervertebral disc degeneration using Pfirrmann criteria [18] (Fig. 1A). Sagittal and axial images were used to assess the presence of a high-intensity signal in the herniation [9] (Fig. 1B) and the herniated disc size. The high-intensity signal in herniated discs was defined as an area on T2-weighted MRI in which the signal and axial intensity exceeded that of the adjacent annulus fibrosus and nucleus pulposus.

Frontal and lateral views on plain lumbar radiography permitted definition of the region of condoliase injection. Lines were drawn on these images between the endpoints of vertebral edges, and the central region was defined as the middle third (Fig. 1C). The bulging ratio of herniation (ratio of the anteroposterior diameters of the herniation and canal space) and the reduction ratio (post- to preoperative bulging ratio) were calculated (Fig. 1D) [19]. The herniated disc area (mm^2^) was measured on sagittal sections between the margins of each pedicle. Reference lines on each section were drawn between the endpoints of the posterior edges of the superior and inferior endplates. Measurement of the areas was performed on a picture archiving and communication system. The herniated volume (mm^3^) was calculated by multiplying the area by the scan thickness (mm) (Fig. 1E) [14, 20]. In all cases, follow-up MRI was performed 3 months after injection to calculate the postoperative bulging ratio and the reduction ratio.

All measurements were made in triplicate by two observers and the average was used. To minimize bias, the observers were blinded to the patient information (e.g., age, gender) during the radiographic measurements. This protocol was implemented to ensure the objectivity of the assessments. To ensure measurement reliability, intra- and inter-rater agreement were assessed using the intraclass correlation coefficient (ICC).

### Statistical analysis

Associations between groups were evaluated by univariate analysis using chi-square and Mann–Whitney U tests. Logistic regression analysis was used to analyze factors associated with efficacy. Univariate analysis was first performed on each covariate. Multivariate logistic regression analysis was then used to identify independent relationships with efficacy for variables with significance in univariate analysis. All analyses were performed using SPSS ver. 23.0 (SPSS Inc., Chicago, IL, USA), with *p* < 0.05 considered to be significant.

## Results

**Fig. 2:**
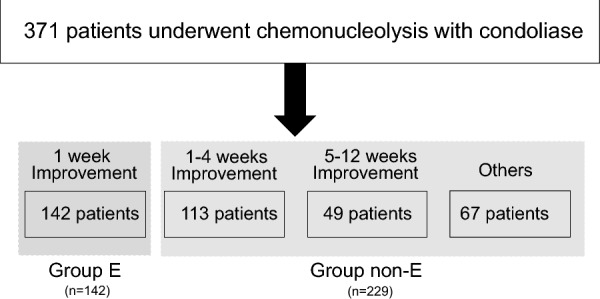
Flowchart of patients in the study. Those defined as “Others” showed no improvement within 12 weeks, including 24 patients who underwent surgery

**Fig. 3:**
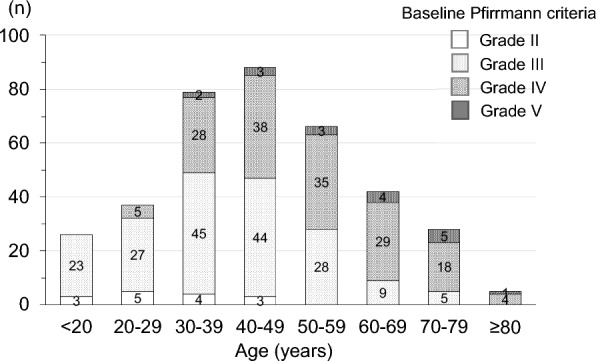
Age distribution of patients divided by Pfirrmann grade

**Fig. 4:**
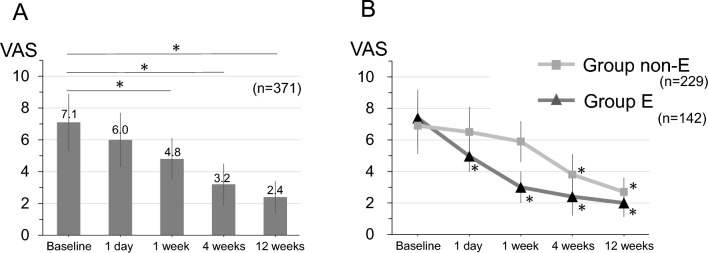
Time course of VAS after condoliase injection. **A**: All patients (*n* = 371). VAS for leg pain improved significantly at 1 week, 4 weeks, and 12 weeks after condoliase injection. *p < 0.05. **B**: VAS in groups E and non-E. *p < 0.05 vs. baseline. VAS: visual analog scale

The subjects were 371 consecutive patients, including 259 males and 112 females with a mean age of 49.9 ± 18.7 years and a mean follow-up period of 13.1 ± 7.4 months. All the patients underwent chemonucleolysis with condoliase, with 142 achieving leg pain improvement ≥ 50% compared to baseline within 1 week, 113 between 1–4 weeks, 49 between 5–12 weeks, and 67 at 12 weeks or more (Fig. 2). Data for the 371 patients were compiled for demographic information and baseline characteristics (Table 1) and age and baseline Pfirrmann grades (Fig. 3). There were no cases with anaphylactic shock or neurologic sequelae. Six patients had rash within 3 days after injection, which was resolved with normal dermatological treatment. The treatment response after condoliase injection is shown in Table 2. Improvement occurred in 21% of cases after one day of condoliase treatment, and in 38% after one week of treatment. The trends in VAS for pain in all cases (Fig. 4A) indicated significant improvements at 1, 4, and 12 weeks after injection. VAS trends in groups E and non-E are shown in Fig. 4B.

**Table 1:** Demographic data and baseline characteristics of the patients (n = 371)

Item	Data
Age (years)	49.9 ± 18.7
Gender (female %)	112 (30%)
*BMI (kg/m*^*2*^*)*	
≤ 18	26 (7%)
18–25	226 (61%)
≥ 25	119 (32%)
*Intervertebral disc level*	
L1/2	8 (2%)
L2/3	15 (4%)
L3/4	19 (5%)
L4/5	185 (50%)
L5/S1	144 (39%)
Symptom duration before injection (months)	7.1 ± 5.8
Follow-up period (months)	13.1 ± 7.4
Baseline Pfirrmann grade	
Grade II	15 (4%)
Grade III	181 (49%)
Grade IV	157 (42%)
Grade V	18 (5%)
High-intensity MRI signal in herniation	70 (19%)
*Classification of herniation*	
Subligamentous	234 (63%)
Transligamentous	137 (37%)
Injection into central region of disc	341 (92%)
Baseline VAS leg pain	7.1 ± 1.8
Bulging ratio of herniation (%)	29.1 ± 10.7
Reduction rate of herniated mass volume (%)	31.2 ± 17.4
≥ 50% decrease of herniated mass volume (n)	96 (26%)

Continuous data are presented as mean ± standard deviation

**Table 2:** Treatment response rate after condoliase administration

Time	Effective (n)	Treatment response rate (%)
1 day	78	21%
1 week	142 *	38%
4 weeks	255 *	68%
12 weeks	304 *	82%

^*^Cumulative sum

**Fig. 5:**
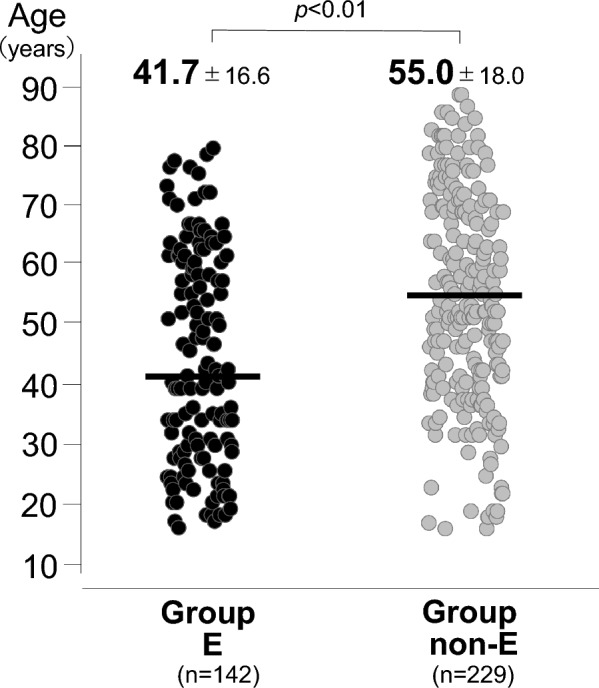
Age distribution in groups E and non-E (41.7 vs. 55.0 years, *p* < 0.01)

In univariate analysis for comparison of group E (n = 142, 38.3%) and group non-E (n = 229), age (41.7 vs. 51.0 years, *p* < 0.01; Fig. 5), baseline Pfirrmann grade (Grade II: 7% vs. 2%, *p* = 0.021; Grade III: 56% vs. 44%, *p* = 0.038; Grade IV: 33% vs. 48%, *p* < 0.01), high-intensity MRI signal in the herniation (30% vs. 13%, *p* < 0.01), and reduction rate of herniated mass volume (38% vs. 27%, *p* = 0.024) were significantly related to early improvement within a week after injection of condoliase (Table 3). In logistic regression analysis, age < 40 years (*p* = 0.022, odds ratio (OR): 1.71, 95% confidence interval (CI): 1.12–4.35), Pfirrmann grade II or III at baseline (*p* = 0.032, OR: 1.86, 95% CI: 1.17–5.41), and a high intensity MRI signal in the herniation (*p* = 0.041, OR: 1.87, 95% CI: 1.06–5.27) were significantly associated with early improvement (Table 4). For radiographic assessment, the ICC for intra-rater reliability was 0.85, indicating high consistency; and the ICC for inter-rater reliability was 0.78, suggesting substantial agreement between observers.

**Table 3:** Comparison of demographic data in patients in groups E and non-E

Item	Group E (n = 142)	Group non-E (n = 229)	*p* value
Age (years)	41.7 ± 16.6	55.0 ± 18.0	< 0.01
Gender (female)	42 (30%)	70 (31%)	0.839
Body mass index (kg/m^2^)	23.3	22.7	0.421
*Intervertebral disc level*			
L1/2	3 (3%)	5 (2%)	0.963
L2/3	5 (3%)	10 (4%)	0.687
L3/4	6 (4%)	13 (6%)	0.537
L4/5	72 (51%)	113 (49%)	0.799
L5/S1	54 (38%)	90 (39%)	0.806
Symptom duration before injection (months)	5.3 [1–13]	8.2 [1–22]	0.027
*Baseline Pfirrmann criteria*			
Grade II	10 (7%)	5 (2%)	0.021
Grade III	79 (56%)	102 (44%)	0.038
Grade IV	46 (33%)	111 (48%)	< 0.01
Grade V	7 (5%)	11 (5%)	0.956
*Classification of herniation*			
Subligamentous	96 (68%)	138 (60%)	0.154
Transligamentous	46 (32%)	91 (40%)	0.154
High-intensity MRI signal in herniation	42 (30%)	28 (13%)	< 0.01
Injection into central region of disc	134 (94%)	207 (90%)	0.172
Baseline VAS leg pain	6.9 ± 1.8	7.3 ± 1.7	0.358
Bulging ratio of herniation (%)	30.2 ± 10.9	28.4 ± 11.2	0.137
Reduction rate of herniated mass volume (%)	38.2 ± 19.2	26.8 ± 14.2	0.024
≥ 50% decrease of herniated mass volume (n)	45 (31%)	51 (22%)	0.044

Continuous data are presented as mean ± standard deviation

**Table 4:** Multivariate analysis of factors associated with early improvement

Item	Odds ratio (95% CI)	*p* value
Age		0.022*
≥ 40	1	
< 40	1.71 (1.12–4.35)	
Baseline Pfirrmann grade		0.032*
Grade IV or V	1	
Grade II or III	1.86 (1.17–5.41)	
Symptom duration before injection (months)		0.214
≥ 6	1	
< 6	0.99 (0.16–5.64)	
Herniation on MRI		0.041*
Without high-intensity signal	1	
With high-intensity signal	1.87 (1.06–5.27)	
Decrease of herniated mass volume		0.145
< 50%	1	
≥ 50%	0.98 (0.42–6.42)	

^*^ *p* < 0.05

### Representative case

**Fig. 6:**
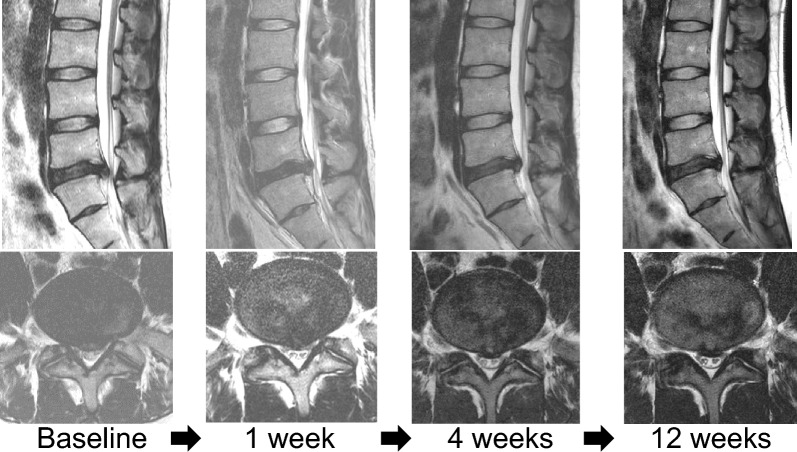
Representative case in group E. L5-S LDH in a 37-year-old male. MRI findings are shown at baseline and 1 week, 4 weeks, and 12 weeks after condoliase injection

Results for a 37-year-old male treated with intradiscal condoliase injection for L5-S LDH are shown in Fig. 6. The patient had suffered left lower-extremity pain for 12 weeks. VAS scores for leg and back pain were 8.4, 4.1, 3.2, 2.4 and 2.0 at baseline, 1 day, 1 week, and 1 and 3 months, respectively. On MRI, migrated herniation (Pfirrmann grade 3; high signal intensity area within the herniation); herniated mass volume, 1220 mm^3^) was reduced 12 weeks after injection (Pfirrmann grade 3; herniated mass volume, 794.1 mm^3^, reduction rate, 35%) (Fig. 6).

## Discussion

Conservative treatment for 6 to 8 weeks is used for LDH, but this condition affects many young to middle-aged people; therefore, faster relief of symptoms to allow an earlier return to work is important for socioeconomic reasons. In our series, 21%, 38%, 68%, and 82% of cases had ≥ 50% improvement in leg pain from baseline at 1 day, 1 week, and 1 and 3 months after condoliase chemonucleolysis, respectively. This study is the first evaluation of the effects of this treatment on the day after injection. Condoliase treatment as a first-line option for LDH has advantages over surgery or conservative treatment based on its cost-effectiveness [21]. Therefore, the choice of condoliase chemonucleolysis before considering surgery is economically appropriate, especially for cases with probable early improvement.

We believe that our series is the largest cohort in which the outcomes of chemonucleolysis with condoliase have been investigated [7, 9–11, 13–15, 19, 22–25] (Table 5), and no large-cohort studies have examined the early therapeutic effects of this treatment. In some cases, symptoms improved rapidly, from immediately after administration to the following day, and the characteristics of these cases are of particular interest. Several factors have been associated with positive outcomes in condoliase therapy, including younger age, shorter symptom duration, herniation at L5/S1, larger herniated disc with significant spinal canal occupancy, notable herniated disc regression, severe disc degeneration, high-intensity signal changes on pretreatment T2-weighted MRI, transligamentous LDH, and absence of prior nerve block treatments [7, 9–15]. In our series, patients with good responses to condoliase treatment had significant improvement in leg pain as early as the day after treatment, with effects lasting for over 3 months. In multivariate analysis, younger patients (< 40 years), baseline Pfirrmann grade, and a high-intensity MRI signal in the herniation were significant predictors of an early response.

**Table 5:** Literature review of chemonucleolysis with condoliase

Author, year, Reference	Number of cases treated with condoliase	Mean age (years)	Sex (M/F)	Follow-up period (months)	Affected level (L1/2,L2/3,L3/4,L4/5,L5/S)	Serious adverse events*/ Allergy-like symptoms**	VAS for leg pain improved ≥ 50% ***	Surgery after injection	Comments
Matsuyama et al., 2018 ^15^	115	39.2 (range NA)	82/33	13	0/0/0/64/51	3/4	84 (73%)	NA	Incidence of adverse drug reactions and decrease in disc height were dose dependent
Chiba et al., 2018 ^7^	81	39.5 (range NA)	50/31	13	0/0/0/45/36	3/4	59 (73%)	8 (10%)	At 1 year after administration, condoliase significantly improved symptoms in patients with LDH and was well tolerated
Nakajima et al., 2020 ^14^	42	47.7 (range NA)	29/13	3.2	0/2/2/23/15	NA	32 (76%)	NA	Most effective in cases with a larger herniated mass volume before treatment; least effective in cases with longer time before treatment
Ishibashi et al., 2020 ^13^	34	32.4 (range 13–68)	24/10	3	0/0/0/25/9	NA	21 (62%)	6 (17%)	Improved leg pain significantly correlated with high-intensity signal change and size of protruded NP
Banno et al., 2021 ^9^	47	48.0 (range 15–81)	27/20	8.2	0/1/2/23/21	NA	33 (70%)	1 (2%)	Efficacy in transligamentous type cases and those with high T2 herniation
Okada et al., 2021 ^11^	82	47.2 (range 18–70)	55/27	9.1	1/5/3/37/36	0/3	70 (85%)	2 (2%)	Condoliase should be injected into the center of the intervertebral disc
Hirai 2021 ^19^	52	45.0 (range NA)	35/17	6.0	1/1/4/30/16	0/2	40 (76%)	3 (5.8%)	Leg pain more likely to improve in patients with high-intensity signal change in the area of LDH
Banno et al., 2022 ^10^	60	44.5 (range NA)	37/23	22	0/1/4/26/29	NA	47 (73%)	8 (13%)	Disc degeneration induced by chemonucleolysis can recover
Kobayashi et al.,2022 ^22^	127	46.6 (range 16–88)	88/39	9.8	2/6/6/66/47	0/3	95 (75%)	16 (13%)	Progression of Pfirrmann grade on MRI significantly associated with improved clinical outcome
Matsuyama 2022 ^23^	228	39.0 (range NA)	115/73	45.8	0/0/0/121/107	NA	NA	31 (13.4%)	Follow-up > 1 year revealed no new safety concerns of condoliase
Banno 2022^24^	67	46.7 (range NA)	44/23	33.5	0/0/4/36/27	0/0	51 (76.1%)	8 (12%)	Chemonucleolysis-induced disc degeneration slightly recovered and maintained for two years post-treatment
Takeuchi 2023 ^25^	101	53.1 (range NA)	80/21	NA	1/3/12/41/44	0/5	88 (87%)	18 (17%)	Calcified or ossified disc herniation may be predictors of unsuccessful treatment

Values are presented as number (%)

^*^ A serious adverse event was any event that occurred during the study period (regardless of its relationship to the treatment) that resulted in death, was life-threatening, required inpatient hospitalization or prolongation of hospitalization for treatment, resulted in persistent or significant disability or incapacity, resulted in a congenital anomaly or birth defect, or caused other clinically significant events or reactions

^**^ Drug eruption and rash

^***^ Baseline vs. 3 months after injection

NA: Not available

Condoliase therapy is based on dehydration of glycosaminoglycans (primarily chondroitin sulfate) on proteoglycans in the nucleus pulposus of the intervertebral disc. This effect may be greater in cases with less intervertebral disc degeneration. However, the initial distribution of condoliase in the disc and the degree of fibrosis in the nucleus pulposus may also be important. Higher injection pressure might be necessary in cases with less degenerated discs, and these discs may be less extensible compared to degenerated discs. Thus, the choice of condoliase injection should be considered carefully in cases with minimal disc degeneration or severely degenerated discs with few proteoglycans in the nucleus pulposus [14].

Symptom improvement following condoliase treatment for LDH is significantly better in younger patients [10, 11, 14], which may be due to higher water content in the nucleus pulposus. A previous series showed that patients aged < 40 years had significantly better clinical outcomes [22, 26]. These findings are consistent with the early improvements after treatment of younger patients, and suggest that disc degeneration, as indicated by Pfirrmann grade II or III, is significantly associated with intervertebral disc degeneration in younger individuals.

Previous studies have found more frequent favorable treatment outcomes when a high-intensity MRI signal is present in the disc [9, 11], and this signal on a T2-weighted image is correlated with discogenic low back pain [27]. Ingrowth of vascularized granulation tissue seen on histological analysis may induce an immune response and lead to recruitment of inflammatory cells in the high-intensity zone [28]. A high-intensity T2-weighted signal in a herniation reflects a hydrated disc and is related to shorter pain duration [27]. Thus, this signal may be predictive for leg pain improvement and may also be a criterion for selecting cases that are likely to respond well to chemonucleolysis with condoliase. Cases with hydrated disc herniation with a high-intensity signal seem to respond better to condoliase, and a change in MRI signal intensity in the herniation is a significant predictor of early clinical improvement.

The protocol was performed at 12 weeks postoperatively. The example case (Fig. 6) underwent lumbar MRI at 1, 4 and 12 weeks postoperatively, but this schedule was not used in every patient. These imaging time points were chosen to monitor the progression of morphological changes in the herniated disc. MRI at 1 and 4 weeks was performed to confirm the immediate effects of the injection and assess any early adverse events in a few cases. This imaging may not be clinically essential for patients with early symptom improvement, but it provides useful data for understanding the early effects of the treatment, which is crucial for refining future protocols.

The limitations of this study include its relatively short follow-up period of 13.1 ± 7.4 months and its retrospective comparative design. Thus, there is a need for evaluation of the long-term efficacy of condoliase injection. Also, early MRI evaluation was not always performed and additional MRI studies are required to visualize the early effects after condoliase injection. There may also be a placebo effect on immediate symptom improvement, although a randomized placebo-controlled trial showed a detectable difference in leg pain improvement between the condoliase and placebo groups only after one week [7]. This suggests that the very early responses seen in this study are due to the therapeutic effect of condoliase. Conservative treatments, such as physical therapy, oral analgesics, or NSAIDs, were continued for some patients after the intradiscal injection of condoliase. These additional treatments, tailored to individual clinical needs, are part of standard care, but may have influenced the observed early symptom improvement. This potential confounding factor could have an impact on the interpretation of the effects of the injection, and this warrants careful consideration in future studies. Regarding the omission of covariance analysis to account for the correlation between age and baseline Pfirrmann grade, older age is known to correlate with higher Pfirrmann grades, and this may have introduced confounding effects that influenced the reliability of the regression results. Finally, the high intra- and inter-rater reliability (ICC = 0.85 and 0.78, respectively) supports the robustness of the measurement method. This level of agreement is consistent with previous studies using similar methods, and this further validates the approach used.

As far as we are aware, this study includes the largest patient cohort treated with chemonucleolysis using condoliase and is the first to identify the characteristics associated with early improvement in this treatment for LDH. Patients want to see improvements in their symptoms as quickly as possible, and this information will be valuable for treatment. As use of condoliase is likely to increase, particularly for relatively young patients, suppression of disc degeneration progression with aging will become increasingly important. Therefore, the impact of disc degeneration progression over the long term, especially in younger patients, should be a focus of longer-term studies.

## Conclusion

The results of this study show that condoliase chemonucleolysis for LDH resulted in a 21% improvement on the next day and a 38% improvement within one week. The study also confirms the safety and efficacy of condoliase chemonucleolysis for treating radicular symptoms in painful LDH, with 82% of patients showing significant improvement without severe adverse events. Age, high-intensity MRI signals, and baseline Pfirrmann grade were significant factors associated with early improvement. Longer-term studies of efficacy and associated factors are needed to identify patients who will benefit most from this treatment.

## Data Availability

No datasets were generated or analysed during the current study.

## Abbreviations

LDH: Lumbar disc herniation
Condoliase: Chondroitin sulfate ABC endolyase
